# Assessing potential brief screening questions for use within different social care‐related contexts to identify individuals experiencing gambling‐related harms: A scoping review

**DOI:** 10.1111/hsc.13976

**Published:** 2022-09-04

**Authors:** Cat Forward, Caroline Norrie, Stephanie Bramley, Heather Wardle, Glenn Stewart, Wesley Dowridge, Steven Nyandu, Jaana Parker, James Shearer, Emily Finch, Jill Manthorpe

**Affiliations:** ^1^ King's College London London UK; ^2^ Department of Health Sciences University of York York UK; ^3^ University of Glasgow Glasgow UK; ^4^ London Borough of Enfield London UK; ^5^ People with Lived Experience (PWLE) Representative BetKnowMore London UK; ^6^ BetKnowMore London UK

**Keywords:** adult social care, gambling‐related harms, health promotion, pathological gambling, public health, scoping review, screening

## Abstract

Gambling‐related harms are increasingly recognised as public health concerns internationally. One response is to improve identification of and support for those affected by gambling‐related harms, including individuals who gamble and those close to them, ‘affected others’. Adult social care services have been identified as a setting in which screening for gambling‐related harms is suitable and desirable. To achieve this, a tool is required which can identify gambling‐related harms experienced by individuals and affected others. This scoping review aimed to identify whether any brief (i.e. three questions or less) screening tools are being used and, if so, how brief screening for gambling‐related harm is being implemented in health and social care‐related contexts. An international English language scoping review of research and grey literature was undertaken between April and July 2021. The search included single‐item and brief screening tools which have been developed to identify gambling‐related harms for individuals and affected others across a range of health and social care‐related contexts. Findings show that screening tools for gambling‐related harms have been developed for use in health settings rather than in social care contexts. For example within gambling, mental health or substance misuse support services. We found no evidence of a brief or single‐item screening tool for identifying harms to individuals and affected others which is of adequate quality to strongly recommend for use in an adult social care setting. Development of a validated brief or single‐item screening tool is recommended to assist adult social care practitioners to effectively screen, identify, support and signpost people affected by gambling‐related harms.

**What is known about this topic**

- Gambling‐related harms are a public health issue.
- Support addressing gambling‐related harms is available, but awareness is lacking.
- Screening can increase signposting to and utilisation of support services.

**What this paper adds**

- Demonstrates that no single‐item or brief screening question/tool for identifying gambling‐related harms experienced by individuals and affected others exists or is being utilised within in an adult social care setting.
- Presents evidence of screening for gambling‐related harms in other settings. This is relevant to social care but needs further context‐specific research.
- Provides evidence that a brief screening tool should be developed and tested for use in adult social care contexts.

## 1 INTRODUCTION

### 1.1 Background

Gambling is recognised by the World Health Organisation as a serious public health challenge (Abbott, 2020). Gambling‐related harms (GRHs) affect adults with care and support needs, impacting on carers, family members, and the public (Public Health England, 2020; Wardle et al., 2019). Multiple harms can be experienced, including debt, mental and physical health impacts, relationship breakdown, increased substance misuse, unemployment, homelessness, theft, and suicide (Elovainio et al., 2017). GRHs differ between individuals but can be grouped into three domains: resources (debt, crime, employment, etc), health (physical health, psychological distress, etc), and relationships (family breakdown, community issues, etc) (Wardle et al., 2018). The Covid‐19 pandemic changed how people gamble but not the extent of public health concern (Griffiths et al., 2020). The UK House of Lords (Select Committee on the Social and Economic Impact of the Gambling Industry, 2020) reported that half of adults in the United Kingdom (UK) gamble at least once a month, including the National Lottery. In England, 400,000 adults are estimated ‘problem’ gamblers, as defined by criteria in the American Psychiatric Association's Diagnostic and Statistical Manual of Mental Disorders (2013). A further 2 million people are at risk of developing a problem with gambling (Wardle et al., 2019). Approximately 7% of the total adult population in Great Britain are negatively affected by another person's gambling, referred to as ‘affected others’, (AOs) (Gunstone & Gosschalk, 2020). AOs are more likely to be women than men, (Public Health England [PHE], 2021) and can experience barriers to support‐seeking such as shame or embarrassment or inaccessibility of local support services (Banks et al., 2018). English Local Authorities (LAs) are being asked to identify and support people experiencing GRHs such as poverty, debt, mental health problems and housing instability (Local Government Authority (LGA)/Public Health England (PHE), 2018). PHE (2021) recommends screening, diagnosis, and treatment of gambling‐related health problems, to enable robust estimates of the costs to the health and social care system.

GRHs disproportionately affect people experiencing social deprivation and ‘vulnerable’ adults who may require support from social care services. However, there are gaps in service provision and evidence addressing this in statutory adult social care (ASC) services (Bramley et al., 2017; O'Dowd, 2019). Screening for alcohol and drug problems is common in ASC assessments but not for GRHs (Galvani, 2015). GRHs can be hidden and often become severe before help‐seeking, exacerbating feelings of stigma and shame (Cowlishaw et al., 2017). Dialogues around responsible gambling can amplify stigma by blaming individual behaviour rather than government and industry regulations (Miller et al., 2018). Affected individuals rarely contact health or care services about GRHs as their presenting condition (Gainsbury et al., 2014). Therefore, screening by ASC services and signposting to gambling support services may improve access to timely support and treatment; allowing ASC services to potentially increase their capacity to support individuals with their other support needs (Reith & Dobbie, 2013; Rogers, 2013).

GRHs reflect and contribute to social inequalities (Thorley et al., 2016). Socially and economically deprived groups are less likely to gamble but when they do are more likely to experience harms related to their gambling (Wardle et al., 2019), as are those with drug and alcohol problems, and people with poorer mental health (Wardle et al., 2016). Cowlishaw et al. (2017) screened patients (*n* = 1058) in 11 English NHS General Practices (primary healthcare) and found that 6% were experiencing GRHs. Research among homeless populations found higher levels of GRHs (around 11%) (Sharman & D'Ardenne, 2018) while research by Citizens Advice has highlighted extensive financial harms due to gambling (Nash et al., 2018).

Local Government Authority/Public Health England (2018) suggests ASC staff can support people impacted by harmful gambling. They advocate training so staff can recognise potential cases and recommend LAs ‘implement screening processes and strengthen data collection’ (p. 26) to reduce the social and economic burden of GRHs. Data are required about the range of GRHs and the resulting costs to LAs.

Furthermore, although social workers in England were supportive of screening, some reported they lacked knowledge about GRHs and lacked confidence in discussing gambling with service users, hence the need for professional development opportunities to improve their ability to support service users (Bramley et al., 2019).

There is little research about screening for GRHs in UK, or specifically, English, social care services (Blank et al., 2021a, 2021b). A systematic review examined screening instruments for gambling disorders in health settings, of 31 tools only three met eligibility criteria (Otto et al., 2020). The authors concluded that few screening instruments have been validated and recommended for use across a large health system. Evidence highlights the importance of developing screens for specific settings and populations, as otherwisem they may not maintain satisfactory levels of sensitivity and specificity (Stinchfield, 2013). Screening tools developed for use in healthcare or population survey contexts are not suitable for use in the social care context due to differences in client vulnerabilities, the differing nature of the interactions and the specific needs of ASC clients. Existing screens do not identify AOs who are eligible for support from gambling support organisations. Therefore, implementing screening across all ASC clients may increase the likelihood of identifying and supporting AOs.

This scoping review was carried out to examine the evidence for brief screening questions already in use and, where possible, to compare their specificity and sensitivity. The wording and the concepts used in the screening questions were also analysed for their acceptability within an English social care context. The scoping review also included a focus on screening for ‘affected others.’

## 2 METHODS

### 2.1 Research question

What is the evidence of brief screening tools (three items or under) being used in health or ASC‐related areas to identify individuals affected by GRHs, and might they be transferable to English ASC contexts?

### 2.2 Study design

A scoping review was undertaken as it was not expected that the literature would be suited to a systematic review (Tricco et al., 2018). The review was conducted using the Preferred Reporting Items for Systematic Reviews and Meta‐Analyses (PRISMA) guidelines, extended for scoping reviews (Moher et al., 2010; Tricco et al., 2018). The study's People with Lived Experience (PWLE) group (*n* = 8) met with researchers to discuss arising findings from the scoping review and three members read draft copies of the article and commented.

### 2.3 Search strategy

Table 1 summarises the search strategy. This was developed using PICo: Population, Phenomenon of Interest; Context (Glasper & Rees, 2016). A search was made of the major databases: Scopus; OvidMedline; PsycInfo; Embase; Web of Science; ASSIA (Applied Social Sciences Index and Abstracts); NHS Evidence, Social Care Online (SCIE), Social Policy and Practice. In addition, the grey literature was searched: GambleAware; GamCare, Gamblers Anonymous; Gordon Moody Association; National Problem Gambling Clinic; King's College London Addictions Department Publications archive; Mental Health Foundation; Open Grey; Society for the Study of Addiction; Public Health Matters; Shelter; Money and Mental Health; Citizens Advice; Stepchange; Carers UK; Centrepoint; St Mungo's (for a definition of grey literature see Benzies et al., 2006). The search was undertaken between April and July 2021.

**TABLE 1:** Summary of search strategy

Population/problem	Phenomenon of interest	Context
Gambler* OR problem gambler* OR moderate‐risk OR low‐risk OR non‐problem OR recreational gambler* OR gambling OR gambling disorder OR disordered gambling OR pathological gambling OR compulsive gambling OR gambling‐related harm* OR gambling related harm* OR harms from gambling OR gambling harm OR problem gamb*	Screening for problem gambling OR routine screening for problem gambling OR Screen* OR one‐item screen* OR brief screen* tool OR screen* measure OR brief intervention OR single‐item OR brief instrument OR one question OR classification instrument OR classification accuracy OR Target question OR Trigger question OR Single question OR Initial question OR Single item OR Probe question OR Asking about OR Asking patient* OR Strategy to identify OR Strategies to identify OR Single screening question OR Simple question OR basic question OR assessment tool OR screening instrument OR routine screening OR assessing gambling OR gambling screening OR brief screening instrument OR gambling harm screen OR starting question OR gateway question OR identif* problem gambling OR PGSI OR GAST‐G	mental health service* OR social care OR social service* OR Primary Care OR addictive behaviour OR public health OR healthcare OR consumer advice OR council OR local government OR local authority OR charity OR housing OR debt OR money advice OR financial OR community‐based OR employee* OR affected other OR loved one OR family OR family member OR friend OR service user OR patient OR care user OR member of the public OR public service
Affected other OR loved one OR family OR family member OR friend OR service user OR patient OR care user OR member of the public	As above	As above

### 2.4 Eligibility

**FIGURE 1:**
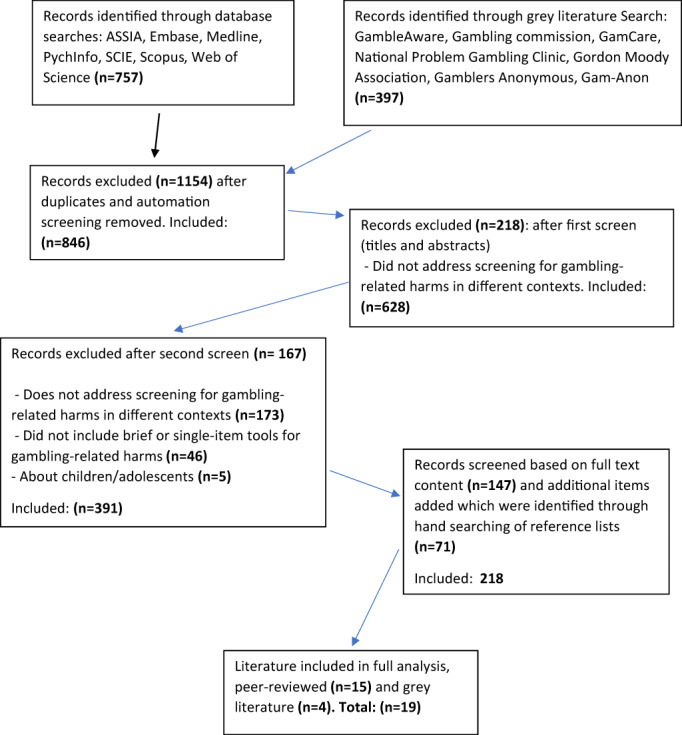
Scoping review flowchart based on a PRISMA flow diagram

Table 2 summarises the inclusion and exclusion criteria. Items were limited to those published between 2007 and 2021 as the 2005 Gambling Act was fully implemented in England, Wales, and Scotland in September 2007, significantly changing and expanding the gambling landscape. As the focus was on ASC, papers dealing with under‐18s were excluded, as were papers examining tools consisting of more than three questions. The choice of three questions was pragmatic. While a single‐item screen is most likely to be easily embedded into a busy ASC setting, so few of the papers had a single‐item screen that it provided very little data for a review of potential question content. Studies including either gamblers or affected others were included, as were contexts such as health or social care, the criminal justice system and addiction support services. As this review was carried out to identify potential screening questions, papers were excluded if they did not provide the wording of the screening question. Hand searching of references was undertaken and additional items were added including from systematic reviews. Figure 1 summarises this process.

**TABLE 2:** Inclusion and exclusion criteria for search

Inclusion criteria	English language Published between 2007 and 2021 Literature concerning individuals aged 18 and over International Literature which presents information about brief (1, 2 or 3 items) or single‐item screening for gambling‐related harms Screening can be in health and social care contexts, addictions, mental health, community, housing, financial advice, and public health Articles were only included where they specifically outlined or discussed the wording of the screening question(s) used Literature about screening populations for gamblers affected by associated harms and affected others (i.e., family members, friends, colleagues)
Exclusion criteria	Not in English language Outside of set publication dates Literature relating to adolescents and children was excluded, that which referred to youth or young people was investigated further. Longer screens i.e., 4 or more item screens Not including screening Not including wording of screening tools

### 2.5 Document selection and appraisal

Papers were removed from the initial search results based on the above criteria. We did not find any papers about screening tools employed to identify those at risk of GRHs, including affected others, within a social care context, therefore wider contexts were included. To improve consistency, 100 papers were reviewed by two reviewers (CN, SB, and CF) independently assessing the same publications. A data extraction table was used to record the selection. Quality appraisal was undertaken for all items included using tools specific to the study designs identified (CASP, 2019). The quality of the papers varied, some being limited by small sample sizes (Hodgins, 2013; Kraus, Etuk, & Potenza, 2020). After assessing for quality and suitability, 15 peer‐reviewed papers and four items from the grey literature were included in the final analysis.

### 2.6 Data extraction

Data were extracted regarding article characteristics (authors, year, methods), screening tool(s) used, context, operationalisation, (e.g. implementation information, method of administration), statistical information (e.g. sensitivity and specificity, where available), benefits and drawbacks of screen used as described, and potential transferability to English ASC contexts. Concepts addressed in the screening questions were also considered such as whether questions measured gambling behaviours or the impacts of gambling.

### 2.7 Data synthesis

We grouped the studies according to whether they related to gamblers or AOs. Data were then synthesised in accordance with the themes from the data extraction table (described above).

## 3 FINDINGS

### 3.1 Search results and document characteristics

Fifteen peer‐reviewed papers, including one systematic review, were included in the final review (Table 3). The papers were from developed countries with the United States (US) (*n* = 5) and Australia (*n* = 4) being the most common, consistent with high rates of disordered gambling in these countries (Harrison et al., 2020; Thomas, 2014). Literature about affected others was scarce and focussed mainly on detailing the nature and extent of GRHs experienced as well as the relationship and demographics of those who identify as affected others (Castrén et al., 2021; Dowling et al., 2014; Landon et al., 2018). From the grey literature, four items or screening tools were identified (Table 4), currently used in services such as primary care (GP surgeries) or criminal justice settings. The definition of GRHs varied between studies with some screens identifying those who are ‘at risk’ and others focusing on those who are ‘severely affected’. Internationally, different types of gambling are popular, and screens may not be comparable in identifying GRHs associated with different forms of gambling.

**TABLE 3:** Summary of key literature

Year	Author(s)	Title	Location	Question used	Setting	Comments on specificity/sensitivity	Method of administration
2009	M. Toce‐Gerstein; D. R. Gerstein; R. A. Volberg	The NODS‐CLiP: A Rapid Screen for Adult Pathological and Problem Gambling	USA	**The NODS‐CLiP:** Have there ever been periods lasting 2 weeks or longer when you spent a lot of time thinking about your gambling experiences or planning out future gambling ventures or bets? Have you ever tried to stop, cut down, or control your gambling? Have you ever lied to family members, friends, or others about how much you gamble or how much money you lost on gambling?	General population field study	Sensitivity—0.96 at identifying problem and pathological gamblers. Specificity—0.95	Researchers: face‐to‐face or telephone
2014	S. Thomas	Problem Gambling	Australia	Have you or anyone in your family an issue with gambling?	GP surgeries	Not addressed	GP: face‐to‐face
2020	I.K. Sørensen, S. Barfod, B. V. Niclasen, U. Becker, L. Penninga & C. V. Lytken Larsen	Prevalence of problems with alcohol, marijuana and gambling among patients in a Regional Hospital in Northern Greenland: investigating the potential for brief interventions in a hospital setting	North Greenland	**NODS‐CLiP:** Have there ever been periods lasting 2 weeks or longer when you spent a lot of time thinking about your gambling experiences or planning out future gambling ventures or bets? Have you ever tried to stop, cut down, or control your gambling? Have you ever lied to family members, friends, or others about how much you gamble or how much money you lost on gambling?	General hospital	Not addressed	Self‐administered questionnaire
2019	P. Sacco; J. J. Frey; C. Callahan; M. Hochheimer; R. Imboden; D. Hyde	Feasibility of Brief Screening for At‐Risk Gambling in Consumer Credit Counselling	USA	**Brief Biosocial Gambling Screen (BBGS):** During the past 12 months have you become restless, irritable or anxious when trying to stop/cut down on gambling? During the past 12 months have you tried to keep your family or friends from knowing how much you gambled? During the past 12 months, did you have such financial trouble as a result of your gambling that you had to get help with living expenses from family, friends or welfare?	Consumer Credit Counselling service	Focus on acceptability in this setting and on comparing prevalence with national estimates	Credit Counselling staff: telephone
2012	M. J. Rockloff	Validation of the Consumption Screen for Problem Gambling (CSPG)	Australia	**Consumption Screen for Problem Gambling (CSPG):** Q1. How often did you gamble in the past 12 months? (Responses—I have NEVER gambled OR I have not gambled at all in the past 12 months = 0 points; Monthly or less = 1 point; 2–4 times a week = 3 points; 4–5 times a week = 4 points; 6 or more times a week = 5 points); Q2: How much time did you spend gambling on a typical day in which you gambled in the past 12 months? (Responses—Less than 30 mins = 0 points; More than 30 mins but less than 1 h = 1 point; More than 1 h but less than 2 h = 2 points; More than 2 h but less than 3 h = 3 points; More than 3 h = 4 points); Q3: How often did you spend more than 2 h gambling (on a single occasion) in the past 12 months? (Responses = Never = 0 points; Less than monthly = 1 point; Monthly = 2 points; Weekly = 3 points; Daily or almost daily = 4 points)	Panel survey (general population)	Found to be consistent with the PGSI. Suggests it may be useful in terms of improving engagement as it addresses consumption instead of impact. Excludes use of lottery and scratch cards.	Self‐administered questionnaire via email
2019	K. Lind; A. H. Salonen; J. Jarvinen‐Tassopoulos; H. Alho; S. Castren	Problem gambling and support preferences among Finnish prisoners: a pilot study in an adult correctional population	Finland	**Brief Biosocial Gambling Screen (BBGS)**	Prison population	Used as a measure of prevalence so not examined.	Self‐administered paper questionnaires
2020	S. W. Kraus; M. N. Potenza; T. Ngo; K. Pugh; K. Bernice; S. D. Shirk	Screening for Gambling Disorder in VA Primary Care Behavioural Health: A Pilot Study	USA	**Brief Biosocial Gambling Screen (BBGS)**	Veterans seeking mental health support	Reliability for the BBGS was acceptable—Cronbach's Alpha = 0.74	Healthcare staff—face‐to‐face
2013	D. C. Hodgins	Reliability and validity of the Sheehan disability scale modified for pathological gambling	Canada	**An interview format version of the Sheehan Disability Scale (SDS) modified to assess gambling treatment outcomes:** To what extent has your gambling problem disrupted your work or studies in the past month on a scale of 0–10, with zero indicating not at all and 10 indicating extremely? To what extent has your gambling problem disrupted your social life in the past month on a scale of 0–10, with zero indicating not at all and 10 indicating extremely? To what extent has your gambling problem disrupted your Family life/household responsibilities in the past month on a scale of 0–10, with zero indicating not at all and 10 indicating extremely?	Participants with pathological gambling in a relapse prevention clinical trial	Internal reliability was estimated at *α* = 0.56. Is intended as a measure of impact on function, not as a screen	Research assistants: telephone
2015	S. S. Himelhoch; H. Miles‐Mclean; D. R. Medoff; J. Kreyenbuhl; L. Rugle; M. Bailey‐Kloch; W. Potts; C. Welsh; J. Brownley	Evaluation of brief screens for gambling disorder in the substance use treatment setting	USA	**Comparison of BBGS, Lie/Bet, NODS‐PERC, NODS‐CLiP**	Intensive outpatient substance use treatment or methadone maintenance services	Accuracy of the brief screens as measured by hit rate were 0.88 for the BBGS, 0.77 for the Lie/Bet, 0.75 for NODS‐PERC, and 0.73 for the NODS‐CLiP. AUC analysis revealed that the NODS‐PERC (AUC: 0.93 [95% CI: 0.91–0.96]) and NODS‐CLiP (AUC: 0.90 [95% CI: 0.86–0.93]) had excellent accuracy	Research assessors—face‐to‐face
2020	G. W. Harrison; M. I. Lau; D. Ross	The risk of gambling problems in the general population: A reconsideration	USA	Examines use of ‘gateway’ questions which use a threshold of frequency of, or expenditure on, gambling	General population survey	Suggests that prevalence is underestimated related to behavioural biases	Survey staff—varied
2008	F. Goodyear‐Smith; N. M. Coupe; B. Arroll; C. R. Elley; S. Sullivan; A. T. McGill	Case finding of lifestyle and mental health disorders in primary care: Validation of the ‘CHAT’ tool	New Zealand	**‘CHAT’ tool:** Do you sometimes feel unhappy or worried after a session of gambling? Does gambling sometimes cause you problems?	GP surgeries	Likelihood ratio of positive test 30.05 (C.I. 17.71–45.81). Likelihood ratio of negative test 0.13 (C.I. 0.02–0.23). Prevalence when compared to gold standard (SOGS) 1.8% (16/909)	Self‐administered paper questionnaires
2018	N. A. Dowling; S. S. Merkouris; V. Manning; R. Volberg; S. J. Lee; S. N. Rodda; D. I. Lubman	Screening for problem gambling within mental health services: a comparison of the classification accuracy of brief instruments	Australia	Compared Lie/Bet Questionnaire, Brief Problem Gambling Screen (BPGS), NODS‐CLiP, NODS‐CLiP2, Brief Biosocial Gambling Screen (BBGS) and NODS‐PERC. The Problem Gambling Severity Index (PGSI) was the reference standard	Various	The five‐item BPGS only tool to identify gambling harms at several levels (low, moderate or problem gambling) (sensitivity = 0.803, specificity = 0.982)). Some identified moderate or problem gambling but not low‐risk gamblers (NODS‐CLiP, three‐item BPGS, NODS‐PERC, four‐item BPGS) (sensitivity = 0.854–0.966, specificity = 0.901–0.954). The two‐item instruments (Lie/Bet and two‐item BPGS) adequately detected problem gambling only (sensitivity = 0.811–0.868, specificity = 0.938–0.943)	Various
2019	N. A. Dowling; S. S. Merkouris; S. Dias; S. N. Rodda; V. Manning; G. J. Youssef; D. I. Lubman; R. A. Volberg	The diagnostic accuracy of brief screening instruments for problem gambling: A systematic review and meta‐analysis	Australia	Meta‐analysis of studies examining Brief Problem Gambling Screen (BPGS‐2), NODS‐CLiP, Problem Gambling Severity Index‐Short Form (PGSI‐SF), NODS‐PERC, NODS‐CLiP2, Lie/Bet Questionnaire and One‐Item Screen	Various	Several showed satisfactory diagnostic accuracy. In terms of single‐item screens, the One‐Item Screen. Studies suggest 92% specificity	Various
2016	G. Challet‐Bouju; B. Perrot; L. Romo; M. Valleur; D. Magalon; M. Fatseas; I. Chereau‐Boudet; A. Luquiens; M. Grall‐Bronnec; J. B. Hardouin	Harmonising Screening for Gambling Problems in Epidemiological Surveys—Development of the Rapid Screener for Problem Gambling (RSPG)	France	**RSPG‐I:** Have you had a gambling practice over the past 12 months? During the past 12 months, have you needed to gamble with increasing amounts of money in order to achieve the desired excitement? During the past 12 months, have you made repeated unsuccessful efforts to control, cut back or stop gambling?	Epidemiological survey	Specificity = 95.2% of participants with a gambling disorder according to the DSM‐5 were identified. Diagnosis efficiency 83%	Designed to be used in interview context
2011	M. J. Rockloff, J. Ehrich, M. Themessl‐Huber, L. G. Evans	Validation of a One‐Item Screen for Problem Gambling	Australia	**One‐Item Screen for Problem Gambling:** In the past 12 months, have you ever had an issue with your gambling?’	Primary care	Sensitivity 21%, specificity 98%	Telephone survey

**TABLE 4:** Grey literature

Year	Source	Title	Location	Question used	Setting	Comments on applicability in social care setting
2020	Gamcare—poster/flowchart	‘Guide for professionals referring adult clients’	UK	In the last 12 months, have you bet more that you could really afford to lose? Or has this happened to someone close to you?	Health or social care	Designed for use in health and social care. Includes affected others
2020	Gamcare—printable information card	NA	UK	Has your gambling or the gambling of someone close to you had a negative effect on your life? *A negative effect might include financial problems, relationship problems or poor health including mental health issues like stress, anxiety or depression*	Prison/criminal justice system	Not intended for oral administration or social care setting but does include broad consideration of effects and affected others. Quite long for a question used to screen in initial contact services
2019	Gamcare—blog post	Working with clients affected by gambling	UK	Is gambling affecting you, or those close to you?	Health or social care	Designed for use in health and social care. Includes affected others
2021	Online screening question used by GP practices	NA	UK	In the last year have you bet more than you could afford to lose? Or has someone in your household bet more than they could afford to lose? *We ask this to understand if problem gambling is affecting your well‐being*	Healthcare	Designed for use in health setting to be completed on a self‐administered online booking form. Includes affected others

A range of screening questions were identified in the literature for identifying individuals affected by GRHs, from single‐item to brief screening tools (up to three questions).

### 3.2 Location and administration of screens

Screening for GRHs is undertaken in contexts such as healthcare settings, GP surgeries (Goodyear‐Smith et al., 2008), substance‐misuse services (Himelhoch et al., 2015) and population‐level surveys (Challet‐Bouju et al., 2016). No studies reported screening tools within ASC, however, within health services, the most comparable of settings, screening for GRHs was both feasible and acceptable (Goodyear‐Smith et al., 2008; Thomas, 2014). This suggests that a screening tool in ASC settings is potentially feasible and merits further research.

The administration of questions varied between face‐to‐face (Kraus, Potenza, et al., 2020), self‐administration using paper questionnaires (Lind et al., 2019) or electronic formats (Rockloff, 2012). The implications of administration method were unexplored but are important given the accelerated move to online services following the Covid‐19 pandemic (Griffiths et al., 2020). When not self‐administered, the screens were undertaken by researchers (Himelhoch et al., 2015), healthcare staff (Kraus, Potenza, et al., 2020), or staff in other settings such as credit counselling services (Sacco et al., 2019). Face‐to‐face interviews may improve engagement (Harrison et al., 2020), while a consideration of the differences in administration by healthcare staff or research staff shows concerns among service users about information sharing among healthcare professionals (Himelhoch et al., 2015). This latter point is relevant to ASC contexts. Service users may be cautious about disclosing gambling issues if they perceive this may impact their entitlement to services.

Grey literature identified tools used in settings such as healthcare and the criminal justice system (Gamcare, 2019, 2020). Some were information cards for display in public areas where the onus is on the individual to self‐refer to support services (Gamcare, 2020). Another item was a blog for health and social care staff, suggesting ways of discussing potential GRHs with service users (Gamcare, 2019). Unlike the peer‐reviewed literature, these items consistently included AOs. However, there was no evidence of their quality, sensitivity or specificity. They were primarily from sources produced by GamCare, a UK charity which provides support and information to those affected by GRHs.

### 3.3 Evaluation of tools compared to gold standard measures

Two papers considered the quality of the screening tools in terms of sensitivity and specificity (Dowling et al., 2018; Toce‐Gerstein et al., 2009), and therefore included several screening tools or questions. It is important to note that tools differed in their measurements of GRHs: some, such as the Lie/Bet screen, a two‐item screening tool (do you bet more than you would like to; do you lie about how much you have bet?) (Johnson et al., 1997), sought to identify only those experiencing a gambling disorder as diagnosable using the DSM‐5 (Diagnostic and Statistical Manual of Mental Disorders—Fifth Edition, American Psychiatric Association, 2013), whereas others such as the five‐item Brief Problem Gambling Screen (BPGS) aimed to identify those who were at risk of GRHs. It is worth noting that differences between the DSM‐IV and DSM‐5 have shifted diagnostic criteria for disordered gambling. This is discussed further elsewhere (Petry et al., 2014). The BPGS illustrates the difficulty in identifying lower risk gamblers with a briefer tool as this can lead to sacrificing specificity for brevity (Dowling et al., 2018).

Dowling et al. (2018) studied mental health service users (*n* = 837) who completed nine brief screening instruments. Patients' responses were then compared to their responses to the nine‐item Problem Gambling Severity Index (PGSI). The five‐item Brief Problem Gambling Score (BPGS) was reported as the most accurate at identifying GRHs. The authors suggested that the two‐item Lie/Bet or two‐item BPGS could be used to adequately detect problem gambling (rather than GRHs) in mental health services which can only accommodate a brief screening tool.

In an international systematic review and meta‐analysis, Dowling et al. (2019) compared the diagnostic accuracy of brief screening instruments for GRHs. The authors compared the evidence about the accuracy of 20 brief screening tools (ranging from one to five items). They concluded that five brief screening tools met the criteria for satisfactory accuracy in detecting both problem and at‐risk gambling. The five‐item BPGS was, again, the most accurate at identifying GRHs however, the present review focuses on identifying briefer screening tools. Dowling et al. (2019) concluded that the Lie‐Bet and One‐Item Screen (Thomas et al., 2010) were promising briefer alternatives, but that more evidence is needed. However, if services needed to administer a briefer screen due to time constraints, then the two‐item Lie/Bet or two‐item BPGS could be employed (see also, Dowling et al., 2018).

Challet‐Bouju et al. (2016) compared the sensitivity and specificity of six screens for GRHs with a sample of 425 gamblers (301 not at risk of GRHs, and 124 affected). These brief tools included Lie/Bet, BPGS, NODS‐CliP (NORC Diagnostic Screen for Gambling Disorders—Control, Lying and Preoccupation), NODS‐CLiP2, Brief Biosocial Gambling Screen, NODS‐PERC, Problem Gambling Severity Index Brief Form (PGSI‐SF), the Case‐finding and Help Assessment Tool (CHAT) and the One‐Item Screen and the Rapid Screener for Problem Gambling (RSPG). The DSM‐5 was used to evaluate the diagnostic efficacy of the Rapid Screener for Problem Gambling (RSPG) which found specificity of 95.2%, a sensitivity of 78.1% and overall diagnostic accuracy of 83% (Challet‐Bouju et al., 2016). This paper also examined the sensitivity and specificity of each question used within the DSM‐5 and the most accurate combinations of up to three questions. This showed that combining questions about gambling with increasing amounts of money (i.e. increasing bets) and loss of control (i.e. making repeated unsuccessful efforts to control, cut back or stop gambling) were the most accurate at indicating what they termed pathological gambling as defined by the DSM‐5.

The Consumption Screen for Problem Gambling (CSPG) considered consumption of gambling products rather than measuring impacts or behaviours. The screen was tested using the PGSI as a gold‐standard measure (Rockloff, 2012). Analysis suggested that the CSPG is consistent with the PGSI and indicates levels of sensitivity of 100%, specificity of 92.7%. The paper concludes that the CSPG can quickly and accurately identify people who are likely to be experiencing GRHs. However, the focus of consumption of gambling products is based on similar tools measuring the consumption of alcohol. It could be argued that consumption in these addictions is not comparable in terms of the way frequency of consumption translates into impact and therefore raises concerns about its use.

The CHAT screening tool was intended for use in the primary care settings for a range of mental health disorders. The GRHs component showed sensitivity of 88% and specificity of 97% when validated against the 20‐item South Oaks Gambling Screen (SOGS) (Goodyear‐Smith et al., 2008). Most other literature used the DSM‐IV or DSM‐5 as the gold standards for identifying problem gambling this was the only paper which used the SOGS for this purpose. This may affect the accuracy of the findings as the SOGS is a less accurate diagnostic guide than the DSM‐5 (Goodie et al., 2013).

The NODS‐CLiP was shown to have levels of sensitivity of 96% and specificity at 95% in identifying problem and pathological gamblers (Toce‐Gerstein et al., 2009). It was derived from the 17‐item NORC Diagnostic Screen for Gambling Disorders (NODS) which in turn is based on the diagnostic criteria set out in the DSM‐IV, suggesting a good standard of accuracy.

In their meta‐analysis, Dowling et al. (2019) re‐analysed data from 25 papers to assess the accuracy of brief screening instruments. The one‐item screen (‘In the past 12 months, have you ever had an issue with your gambling’?), was initially shown to be promising. It showed 92% sensitivity and 96% specificity against a gold standard (PGSI) and the authors concluded it could be used in health services or research. However, it was developed for use in a primary care setting, does not screen for affected others and has limited and variable evidence regarding its application in clinical settings (Dowling et al., 2019; Thomas et al., 2010). Further assessment suggests that the addition of a timeframe, for example asking ‘In the past 12 months, have you ever had an issue with your gambling’? may affect the accuracy of the one‐item screen resulting in high levels of false negatives (79%) (Rockloff et al., 2011). The authors questioned if this could be attributed to issues around the lack of willingness of participants to admit GRHs, this is likely to be the same with any tool and therefore something which needs to be factored into the development of future screens.

Unlike other screens, the one‐item screen suggested in a later paper by Thomas (2014) does include affected others (‘Have you or anyone in your family had an issue with gambling’?), however, this paper does not assess the accuracy or validity of this question and differs in wording from the one‐item screen previously tested by the same author which does not include affected others (Thomas et al., 2010).

There was limited evidence regarding brief screening tools being used to identify AOs and no evidence of a gold standard for such a screen. This limits health and social care services in identifying AOs at risk of experiencing GRHs. The grey literature identified screens developed by GamCare, a UK gambling support charity, (Gamcare, 2019, 2020) where screening questions were made relevant to affected others by adding ‘or someone close to you’ or ‘someone else’. However, there is no evidence about which statutory services are administering these screens, their acceptability to staff and service users, or their accuracy at correctly identifying AOs.

### 3.4 Transferability of identified screens to ASC settings

The screening questions and approaches outlined here were unable to demonstrate suitability for a social care setting as they were administered in epidemiological surveys (Challet‐Bouju et al., 2016), criminal justice contexts (Lind et al., 2019), or primary care settings (Rockloff et al., 2011). In addition to this, many focused on identifying only the disordered gambler rather than including AOs. Many of the longer screens such as the five‐item BPGS would potentially be too long to be administered within all ASC assessments context when working with the time constraints and competing service demands (Dowling et al., 2019).

The commonly used Lie/Bet screen is examined in several papers included in this review (Dowling et al., 2018, 2019; Himelhoch et al., 2015). It is, however, diagnostically focused and may not capture the wider range of GRHs. This may limit its applicability in social care settings where ASC staff's professional responsibility is to ensure users' well‐being and safety.

The grey literature shows that some GP surgeries in the UK are using a screening question regarding GRHs as part of their online booking services (see Table 4). This approach is more transferable to ASC settings, due to its brevity and its inclusion in a broader health and well‐being context. It may also be relevant to social care settings as initial contacts may be online. This is an amended version of the Lie/Bet screening tool and aims to capture affected others as shown in the language used (i.e. ‘Or has someone in your household bet more than they could afford to lose’?).

Behavioural biases related to stigma, and variations in the design of screening tools were highlighted as factors which are likely to result in underreporting of GRHs (Harrison et al., 2020). These are both relevant to an ASC context but relate more broadly to population level surveys. Evidence indicates that screening programmes in a range of settings may improve identification of those who are at risk of GRHs by encouraging people to overcome reluctance to discuss the subject (Sacco et al., 2019). In turn, it is expected that this would lead to an increased uptake of support services which have been shown to be effective (Thomas, 2014). By screening for GRHs across a range of health and social care settings, this may increase opportunities for those at risk to be identified and offered support.

## 4 DISCUSSION

Brief screening tools are in use for identifying individuals experiencing GRHs across several contexts. Healthcare settings or general population level surveys are the most common contexts. This review has revealed a lack of evidence regarding the feasibility of introducing these brief screening tools in ASC contexts. Many of the screens examined are truncated versions of longer, diagnostically focussed assessments and therefore screen for disordered gambling. This focus on clinically diagnosable harm excludes those at risk of GRHs, including affected others who can benefit from support services. There is evidence that staff and service users found screening questions acceptable within the context of broader health and well‐being assessments, through either online or face‐to‐face administration (Blank et al., 2021b; Kraus, Potenza, et al., 2020).

An evaluation of a gambling support initiative in Citizens Advice (CA) services provides some evidence regarding implementation. The GambleAware General Screening Tool (GAST‐G) (a four‐item screening tool for identifying individuals affected by GRHs and affected others) was used in CA practice to assess the feasibility of the tool being embedded into routine practice (Kantar Public, 2021). The evaluation highlighted that in addition to the screening tool, consideration should be given to staff in terms of training, their capacity to screen (e.g. time required), their confidence in supporting gamblers (e.g. signposting to additional support), and at which point screening for gambling harm takes place (e.g. during initial telephone/online assessments or face‐to‐face meetings) (Kantar Public, 2021). Staff capacity is an important consideration in busy services such as CA or ASC contexts as this may have repercussions if high levels of unmet need are identified by screening programmes. More data are needed to assess the extent of this issue. The CA report also highlighted some clients' reluctance to disclose gambling behaviours because of perceived and felt stigma, a point made in other studies and which is an ongoing challenge in this field.

In terms of implementing screening in an ASC setting, Guilcher et al. (2020) undertook a mapping exercise with 30 health and social care professionals in Canada. Forty‐five statements were identified when envisaging embedding a screening tool within a service. These were categorised into five clusters: top level (e.g. ‘buy‐in’ from senior management), screening tools (e.g. practitioners wanted more knowledge of the range of screening tools available); up‐skilling and training; integration of screening into current workloads; team resources and support (e.g. having an in‐house champion and dedicated time).

Our scoping review has indicated a lack of evidence regarding brief measures to screen for GRHs. Using the PGSI as a gold standard, brief screening tools such as the two‐item BPGS were shown to have only adequate sensitivity and specificity (Dowling et al., 2018). While the one‐Item screen performed well against other screens with 92% specificity and 95% sensitivity, this had insufficient evidence in a range of settings and across timeframes, and there is a suggestion that adding a timeframe of 12 months significantly decreased its sensitivity (Dowling et al., 2019; Rockloff et al., 2011). This suggests that more needs to be done to establish which brief measure is effective at identifying GRHs, particularly when they are employed in ASC settings.

There are few screening tools developed specifically for AOs. A six‐item Problem Gambling Significant Other Impact Scale (PG‐SOIS) has been developed (Dowling et al., 2014), however, this focused on measuring harms *after* the person has been identified as an AO. Evidence tended to focus on the demographics of AOs; the harms that they experienced and their relationship to the gambler, rather than ways of identifying them as individuals who potentially need to be signposted for support. This highlights the need for evidence of screening in this population (Castrén et al., 2021; Landon et al., 2018). It is suggested that implementing screening for AOs as part of their contact with health or social care support services could improve the likelihood that they would seek support, given that help‐seeking rates are low among AOs (Castrén et al., 2021).

Given the growing calls for a public health approach to GRHs, work is ongoing to fully understand the extent to which gambling is a public health issue, which people are affected, and the extent of harms experienced (Public Health England, 2021b). There are also calls for interventions across the gambling ‘pathway’ as these may be more likely to be effective in addressing GRHs (Blank et al., 2021a). Therefore, existing literature can inform the introduction of screening in ASC contexts, as evidence suggests that screening is acceptable when it is included as part of the initial assessment procedure in GP surgeries or general hospitals (Sorensen et al., 2020; Thomas, 2014).

This review indicates that some brief screening tools in use have been assessed for sensitivity and specificity and, although some promising results, there is not currently a tool which has been shown to be performing better than the alternatives. There is a lack of evidence regarding the use of such a tool in an ASC setting, including one which screens for both AOs and disordered gamblers. This suggests a need to develop a brief screening tool, ideally a single‐item screen, which could be assessed for use within an ASC context to identify those at risk of GRHs.

### 4.1 Recommendations

There is currently no brief or single‐item screening tool which has been identified as being suitable for use in an ASC context for identifying individuals and affected others experiencing gambling‐related harm. There is a particular lack of evidence regarding a single‐item screen which is likely to be feasible and acceptable for implementation within the ASC assessment context. It is therefore recommended that a single‐item screen be developed for use in ASC settings and evaluated for its efficacy.

### 4.2 Limitations

This review sought to identify and outline the literature around brief screening tools for potential implementation within a social care context. It may be limited by the inclusion of only English‐language papers. In addition to this, the review was limited by the availability of studies, particularly regarding affected others.

## 5 CONCLUSIONS

While there is evidence to suggest that screening for GRHs experienced by gamblers and affected others within adult social care contexts are both necessary and feasible, there is little evidence evaluating current practices. This scoping review has examined existing evidence regarding brief or single‐item screening tools which have the potential to be used in ASC settings. We found no evidence of screening items developed specifically for this setting. Alternative tools are available, and several have been evaluated in terms of their specificity and sensitivity, however, most of the existing screening tools address widely recognised concepts related to GRHs such as behavioural aspects or harms and impacts on daily life. The available tools currently have limited evidence of satisfactory levels of specificity and sensitivity, and none were tested in an ASC setting. Few of the potential screening tools identified included screening for affected others, despite evidence that is a group for whom support would be beneficial. This indicates the need for the development and evaluation of the implementation of a brief screening tool which can be used in an ASC setting which screens for those experiencing GRHs because of their own or someone else's gambling behaviour. The authors are currently undertaking work developing and testing a question which can be used to identify people affected by gambling harms in adult social care.

## AUTHOR CONTRIBUTIONS

Cat Forward reviewed retrieved articles, read selected literature, undertook analysis of identified literature and drafted this paper. Stephanie Bramley and Caroline Norrie developed the search strategy, reviewed retrieved articles, created the Data Extraction Table, read selected literature and reviewed drafts of this paper. Thanks to Steven Nyandu, Wesley Dowridge and Jaana Parker who provided guidance and feedback from a lived experience perspective and commented on earlier drafts of this paper. Stephanie Bramley, Caroline Norrie, Glenn Stewart, Heather Wardle and Jill Manthorpe commented on the drafts of this paper. Database searches were by KCL Library services.

## CONFLICT OF INTEREST

CN, SB, JM and HW have all received research funding from GambleAware (GA), a national charity mandated by government to fund research into gambling‐related harms. GA receives funding through voluntary donations from the gambling industry but decisions about what research to fund and research questions are made by the UK Gambling Commission, the industry regulatory, and are informed by the needs of the Department for Digital Culture Media and Sport. GA also funds the national treatment network for gambling‐related harms of which GamCare is a part. CN, SB and JM have received research funding from GamCare. SB was previously employed by Citizens Advice Calderdale as a Gambling Support Service Trainer following GambleAware's partnership with Citizens Advice. HW is Deputy Chair of the Advisory Board on Safer Gambling, which provides advice to the Gambling Commission on gambling policy and is remunerated by them. She runs a research consultancy which provides research services to public and third sector parties. She does not nor has not provided research or consultancy services to industry. She leads the Gambling & Place Hub at Geofutures which also only works for public and third sector funders. EF is a Trustee of GambleAware.

## Data Availability

The datasets generated and analysed as part of this study are available from the corresponding author on reasonable request.
